# Protocol of Pakistan randomized and observational trial to evaluate coronavirus treatment among newly diagnosed patients with COVID-19: Azithromycin, Oseltamivir, and Hydroxychloquine

**DOI:** 10.12669/pjms.38.5.5512

**Published:** 2022

**Authors:** Shehnoor Azhar, Javed Akram, Muhammad Shahzad, Waqas Latif, Khalid Saeed Khan

**Affiliations:** 1 Dr. Shehnoor Azhar, BDS MPH. Assistant Professor Public Health, Doctoral candidate in Public Health, Silk Road Clinical Research Center, University of Health Sciences Lahore, Pakistan. Dept. of Public Health, University of Granada, Spain; 2 Prof. Javed Akram, FRCP, MBBS. Vice Chancellor/Professor of Medicine, University of Health Sciences Lahore, Pakistan; 3 Muhammad Shahzad, PhD. Head of Pharmacology, University of Health Sciences Lahore, Pakistan; 4 Waqas Latif, M.Sc. Data Analyst, Quality Enhancement Cell, University of Health Sciences Lahore, Pakistan; 5 Khalid Saeed Khan, MBBS MRCOG. Distinguished Investigator at University of Granada funded by the Beatriz Galindo, (Senior modality) program of the Spanish Ministry of Education, Department of Clinical Medicine & Public Health, University of Granada, Spain

**Keywords:** SARS-CoV-2, Hydroxychloroquine, Azithromycin, Oseltamivir, Multi-center, Adaptive, Randomization, Health, SARS-CoV-2, Off-label

## Abstract

**Background & Objective:**

This study aimed to assess the clinical effectiveness of Hydroxychloroquine Sulfate (200 mg orally 8 hourlies thrice a day for 5 days), oseltamivir (75 mg orally twice a day for 5 days), and Azithromycin (500 mg orally daily on day 1, followed by 250 mg orally twice a day on days 2-5) alone and in combination (in seven groups).

**Methods & Analysis:**

An adaptive design is deployed, set within a comprehensive cohort study, to permit flexibility in fast-changing clinical and public health scenario. Primary outcomes include turning the test negative for coronavirus nucliec acid and in bringing about clinical improvement on day 7 of follow-up on a seven-point ordinal scale. The randomized study will recruit participants of either gender above 18 years of age who will test positive for SARS-CoV-2 on Quantitative Reverse Transcription Polymerase Chain Reaction (PCR). Pregnant or lactating females, and those with severe respiratory distress, or with serious comorbidities will be excluded. Randomization will be done maintaining concealment of allocation sequence using a computer-generated random number list. The sample size will be subjected to periodic reviews by National Data Safety and Monitoring Board.

**Ethics and Dissemination:**

The trial is approved by the National Bioethics Committee (No.4-87/NBC-471-COVID-19-05/20/) and institutional Ethical Review Committee. This clinical trial conducted under Good Clinical Practice is expected to inform patients clinical guidelines for the use of these drugs in newly diagnosed with SARS-CoV-2.

**Trial Registration:**

The trial was prospectively registered on April 08, 2020 at clinicaltrials.gov with ID: NCT04338698

## INTRODUCTION

Initial warnings that all countries would suffer SARS-COV-2 (COVID-19) pandemic in 2020 have come true.^1^,^2^ With mean incubation period reported around 6 to 7 days^3^, the disease is characterized by fever, sore throat, dyspnea, loss of smell and taste that could progress to irreversible damage to lungs leading to death.^4^,^5^ The global fatality rate of 3 – 4% has been estimated.^6^,^7^ In the continued absence of a definitive treatment or vaccine, this pandemic continues to harm public health, lifestyles, and economies around the world.^8^

Hydroxychloroquine Sulphate (HQ), commonly used against malaria and rheumatoid arthritis in Indo-Asian region, was reported to stop symptomatic progression of SARS-Cov-2 by inhibiting pH dependent steps of viral replication.^9^,^10^ It’s effectiveness as monotherapy or in combination with common antibiotics and antivirals is being evaluated around the world.^11^ Nonrandomized study of Guatret et al. promoted the use of macrolides like Azithromycin with HQ in clearing the virus at day 6 among hospitalized patients.^12^,^13^ None of the 21 patients enrolled in this study reported any adverse drug reactions. Recruitment in HQ arm of ongoing Solidarity Trial resumed after initial concerns of cardiotoxicity were addresses by data safety review.^14^,^15^ To date, several countries have seen widespread compassionate use of HQ and Azithromycin during the ongoing pandemic.^6^,^12^,^16^

Ulrich and Pillat have attributed interest in common antibiotic Azithromycin (Macrolide) to its mechanism of action against rhinovirus - interference with ligand/CD147 receptor interactions and decrease expression of some metalloproteinases (downstream to CD147) that inhibit viral replication.^6^,^17^,^18^ Oseltamivir was one of the earliest drugs tested on SARS-COV-2 patients in Wuhan, China.^19^ With proven effectiveness against influenza viruses, it will be evaluated as monotherapy and in combinations to rule out sharing of the same neuraminidase enzyme by novel Coronavirus.^18^

## OBJECTIVES

### 2.1 Primary Objective:

- To evaluate the effectiveness of Hydroxychloroquine Sulfate (200 mg orally 8hr thrice a day for 5 days) versus oseltamivir (75 mg orally twice a day for 5 days) versus Azithromycin (500 mg orally daily on day 1, followed by 250 mg orally twice a day on days 2-5) as monotherapy and in various combinations in clearing the coronavirus nucleic acid from throat and nasal swab and in bringing about clinical improvement on day 7 of follow-up (primary outcomes).

### 2.2 Secondary Objectives:

- To evaluate various parameters like PCR and clinical status on extended follow-up, self-reported quality of life, and symptoms scores, within all randomized participants stratified by subgroups
- To follow up participants not consenting to randomization for the outcomes of SARS-Cov-2 infection with supportive treatment only

### 3 Study Design:

An adaptive design, set within a comprehensive cohort study, is chosen because it permits flexibility in this fast-changing clinical and public health scenario.^20^ The randomized study will be a multicenter, multiarm, multistage, randomized controlled trial with a parallel design.

### 4 Setting:

Study sites comprise major public-sector tertiary care health facilities and COVID-19 field hospitals being setup within province of Punjab. Hospitals in other provinces and federal areas will also be invited for participation. Households with self-isolating individuals could also participate in the study.

### 5 Eligibility Criteria:

Eligible will be newly diagnosed patients without any comorbidities or those with controlled chronic medical conditions, e.g., diabetes mellitus and hypertension. Participants of either gender and above 18 years of age, having tested positive for COVID-19 on PCR, will be invited. Participants who are pregnant or lactating, already taking any treatment, under respiratory distress or severely dyspneic, have liver and kidney failure will be excluded from the study. Each participant will undergo baseline investigation i.e., liver function tests, renal function tests, urinalysis, and Complete Blood Count; at the time of enrollment. However, diagnostic facilities and hospital admission criteria could vary across cities under the circumstances. A case report form (CRF) has been developed for this proposal and attached at the end of this document.

### Inclusion Criteria:

1. Confirmed SARS-CoV-2 (COVID-19) infection by a positive test result or not hospitalized (clinical scale score 1 - 2).
2. Either gender, and over the age of 18 years
3. Symptomatic for example fever, dry cough, myalgias

### Exclusion Criteria:

1. Confirmed absence of SARS-CoV-2 (COVID-19) infection by a negative test result.
2. Have uncontrolled or drug-dependent chronic conditions such as heart disease, liver and kidney failure.
3. Ongoing respiratory distress or severely dyspneic add after the existing sentence (Clinical scale score 4 and above).
4. Pregnant or currently lactating female.
5. Immunocompromised, atopic and/or systemic disease(s).
6. On other antiviral drugs.
7. History of allergy to any of the drugs to be administered in this study.

## METHODS

Both recruitment and administration of study drugs will be carried out by any member of the site investigation team. All site teams have been trained accordingly. Participants will be divided into seven intervention groups (A to G) as per given Flow Chart). Study physician(s) will administer three drugs as monotherapy (to three intervention arms – A, B, C), in combination regimen of any of the two trial drugs (to three additional arms – D, E, F), and seventh intervention arm comprising all three drugs (G). The three drugs with respective dosages are - Hydroxychloroquine Sulfate (200 mg orally 8hr thrice a day for five days) versus oseltamivir (75 mg orally twice a day for five days) versus Azithromycin (500 mg orally daily on Day-1, followed by 250 mg orally twice a day on days 2-5).

Study physicians will observe, interview, and report any suspected adverse drug reaction (sADR) to their unit teams and subsequently to electronic data entry module. “A sADR is an unwanted or harmful reaction experienced following the administration of a drug or combination of drugs under normal conditions of use and is suspected to be related to the drug”. In case of sADR, the participant will be informed of discontinuation of given therapy until resolution of symptoms is observed and/or receipt of corresponding investigations (Electrocardiogram, Hepatotoxicity, Nephrotoxicity, Myelotoxicity) confirm the suspicion or otherwise. Findings will be conveyed to participant and recorded on electronic data module. A written report of any confirmed episode will also be immediately submitted to Drug Regulatory Authority of Pakistan (DRAP) on its prescribed proforma (attached with email as separate documents). Any decision to modify the study protocol or drug dosage will be informed by aggregated data on ADRs and Drug Regulatory Authority is a statutory forum to exercise these decisions (if warranted) through pre-notified National Data Safety & Monitoring Board (NDSMB). Irrespective of sADR, the study participants reserve the right to withdraw their informed consent at any point of time throughout recruitment period.

Administration of drugs to participants will be directly observed by the clinical staff at the study sites. Each of the site team has been trained to maintain a record of pharmaceutical supplies from stock entries to disposition. All supplies are labelled NOT FOR SALE from the source. Sites will be responsible to project demand corresponding with the rate of recruitment, monitor appropriate usage, and maintain a folder of used drug packaging for site audits.

The site investigators are responsible to exercise their clinical judgement in provision of best diagnostic and therapeutic care to enrolled patients beyond this protocol, and record each/any of those within patient file and electronic data entry module.

### Study Measures:

Investigators will ensure collection of nasopharyngeal and oropharyngeal swab samples from participants at days 3, 5, and 7 (those positive at day 7 will be followed up further till day 14 and possibly beyond) for monitoring of primary endpoints or outcomes – time to turn negative for COVID-19 on PCR and clinical status on the 7-point ordinal scale. For hospitalized participants, baseline investigations such as CBC, Urea electrolytes, kidney (Creatinine), liver (Liver Function Tests), and cardiac functions (Enzymes, D-dimers, ECG) are to be recorded preferably at the time of admission to hospital and as frequently as permissible within given resources. All participants should have undergone these investigations at least once during the recruiting period. Diagnostics such as Electrocardiogram, Hepatotoxicity, Nephrotoxicity, Myelotoxicity are indicated immediately upon suspected ADR as explained on Page 8 (Methods). It must be mentioned that clinical and diagnostic facilities for COVID-19 vary across study sites due to administrative and geographical factors known to shape emergency responses. A supplementary file (CRF.docx) has been attached to the submission as Case Report Form.

Secondary outcomes like Quality of Life and Symptoms Score will be recorded on day 0 and 7 using standardized tools such as World Health Organization Quality of Life Questionnaire (WHO QoL) and Wisconsin Upper Respiratory Symptoms Survey (WURSS) respectively (attached with email as additional documents). The former is expected to capture participant’s lived experience during care that has not been documented in literature to date. The latter is expected to indicate any respiratory co-infection including those acquired from the hospital. The investigators will also monitor and record variables including duration it takes to oxygenate and ventilate, duration stayed oxygenated and ventilate, number of hospitals stay days, admission to intensive care, and mortality. This methodology has been peer reviewed by an external expert (Annex II). A GCP certified, experienced researcher will conduct the training of up to 50 investigators across 13 participating sites to prepare them for all aspects of this study.

### Sample size:

This is an adaptive design and parameters for formal sample size calculation in a new disease of a previously unknown virus are not available.^21^ Thus, the final sample size will be subjected to periodic reviews at each stage of adaptive design and subsequent advice of National Data Safety and Monitoring Board (NDSMB) notified by Drug Regulatory Authority of Pakistan. Recruitment will end upon the advice of NDSMB.


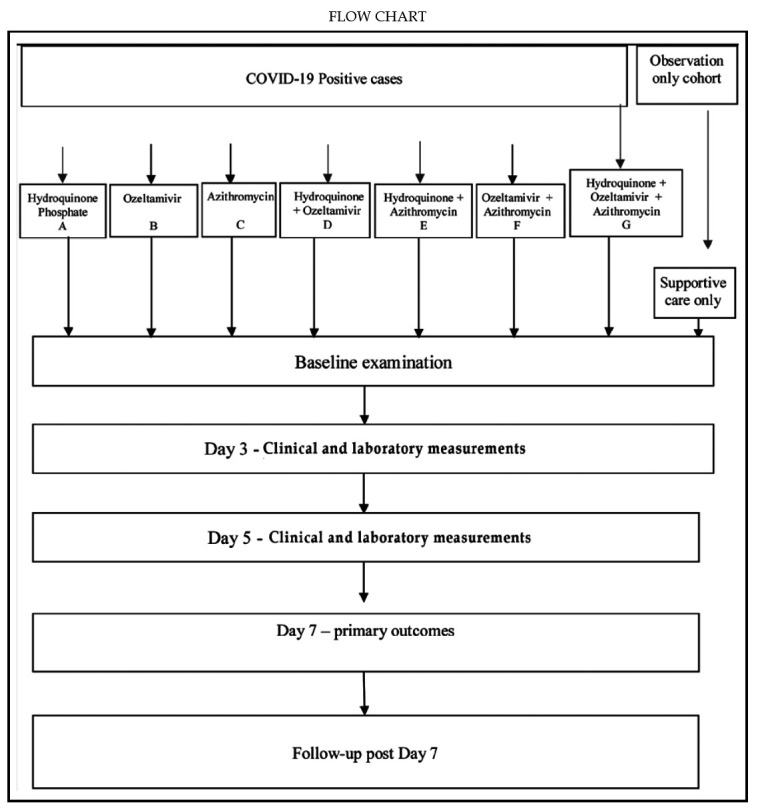


For a standard two-arm, head-to-head trial, the sample size would have been as follows: in order to detect a difference in the laboratory-based primary outcome of 10% (assuming a 50% of participants receiving Hydroxychloroquine Sulfate turn test negative at day 7 and expected rates in comparator groups of 60%), at a significance level 5% and a power of 90%, authors propose the theoretical sample size of approximately 520 subjects in each group. This sample size will be enough to detect a small-medium difference (Cohen’s d 0.3, a significance level 5% and a power of 90%) in the clinical primary outcome as the clinical situation of the pandemic stands. Planned blinded sample size re-estimations will need to be undertaken in which we will constantly re-examine the proposed sample size assumptions. We will aim to maintain the trial power at 90% (established through interim analyses being conducted by NDSMB) even if the proposed sample size assumptions turn out to be far from the observed data. The adaptation at each stage will allow us to drop out fewer promising interventions and will prevent an underpowered trial for the most promising interventions. Recruitment will end upon advice of NDSMB. As the planned modifications will be undertaken in a blinded fashion, there will be no increase in the type I error rate.

### Recruitment:

All patients fulfilling the eligibility criteria and providing informed consent are eligible for this study. Going by the projections, it is expected to enroll substantial number of participants from clinical facilities. A coordination group has been created on digital platform for immediate response to queries, learning from each other’s experiences, and continuity of operations. However, if health system indicates creep, investigators could recruit patients self-isolating at homes. Upon identification by site investigators in the hospital, this cohort would be contacted by trained researchers and data collectors via phone and if informed consent is obtained, recruited. The allocated treatment medicines for 14 days would be delivered. Similarly, swab samples and other clinical information would be collected from participants’ homes in case they cannot travel for testing. All other clinical data would be collected via phone and entered directly into the IT module. The unused or used drug packaging will be collected from participants’ homes upon completion of recruitment period of testing negative on PCR whichever comes first. It is expected that a wider pool of both hospitalized and home-isolated patients would be enough to meet the estimated sample size within the given timeframe.

### Randomization:

Participants will be randomized, maintaining concealment of allocation sequence, using a computer-generated random number list of variable block size into multiple intervention groups in the allocation ratio of 1:1 for all groups (and an observation only group will emerge from those not consenting to randomization). Stratification for age will be used initially to ensure that groups remain balanced in size and prognosis.

Randomization sequence was created by study biostatistician using Sealed Envelope Ltd 2019 and was stratified by study center and age using random block sizes of 28 and 42 sizes into seven treatments.^11^ Before randomizing, recruiting physician will confirm eligibility and obtain written informed consent from the participant. Upon entering participant’s demographic details in the IT module, a unique patient ID for the said center and treatment arm would be allocated automatically meaning the recruiting investigator will have no control over assigning intervention.

### Data Collection, Management and Analysis:

The COVID-19 Patients Research Portal (IT Module) is an electronic case reporting form (CRF) developed by IT Department of University of Health Sciences, Lahore and accessible via theprotect.com.pk. It is expected to fully facilitate both investigators as well study participants. Using a computer desktop or any portable device such as personal computers and tablets, it allows input of data directly from patients’ bedsides for facilities that are electronically equipped and offer reliable internet within isolation areas. It was finalized after pilot testing. Considering the highly contagious nature of this disease, the electronic CRF is expected to minimize cross-infection possible with use of printed study tools. The IT module has the option for investigators to withdraw a case should a participant wishes to discontinue enrollment in the study at any point of time.

### Data Entry:

The role of IT Manager has been assigned to any member of the site team or his/her nominee. (S)he will have exclusive access to the data entry module under given terms of use to ensure data protection and privacy. Data will be entered immediately upon receipt. Sites are trained to develop a housekeeping system to keep track of local data including date stamping all documents on receipt. For data completion and validation, consistency checks would be performed during entry and warning(s) (if any) will be displayed. Data will be copied on a different server regularly. In case of an erroneous entry, the IT Manager will contact designated IT resource at UHS or Lead Biostatistician, provide the justification and get the issue resolved. Each of such correction requests would be recorded.

### Analyses:

All trial data will be analyzed by biostatistician based at the University of Health Sciences, Lahore using an a priori, approved analysis plan.^22^ Interim analyses at each stage (as per advice from NDSMB) of the adaptive design will be pre-planned and undertaken confidentially keeping investigators and participants blind till the end of the trial. Outcomes will be analyzed in accordance to the group in which the participants were randomized deploying the intention-to-treat principle. The demographic characteristics will be presented using descriptive statistics; normally distributed data by the mean and standard deviation (SD). Binary and categorical variables will be presented using counts and percentages. All inferential statistics will be reported at alpha level of 0.05 corrected for the presence of two primary outcomes. The primary analyses are presented in the following:

### Primary analyses for outcome 1

- PCR positivity or negativity will be presented as counts and percentages for all seven study groups on Day 7. Chi-square will test the hypothesis.

### Primary analyses for outcome 2

- The clinical progression of disease will be presented as counts and percentages for all seven study groups on Day 7. Clinical improvement on 7-point ordinal scale will be captured by reduction in score.

### 3. Other analyses

All other analyses will be hypothesis generating as presented below

- Data on PCR positivity or negativity on Day 14
- Clinical status on day 14 according to the 7-point ordinal scale

Interim analyses will be conducted without disclosing groups, thereby maintaining a level of blinding in interpretation of interim results. The confidential interim analyses at each stage of the adaptive design will examine for imbalance in co-morbidity at baseline. If any differences are observed then stratification may be modified confidentially.

Secondary analyses will deploy multiple regression models as well Chi square, Fisher’s exact test and one-way ANOVA to compare groups. The two-sided p-value less than or equal to 0.05 will be taken as significant. Imputation method will be used to handle missing data.

### Data safety and monitoring:

The independent National Data Safety and Monitoring Board (NDSMB) has been notified by Drug Regulatory Authority of Pakistan to oversee all COVID-19 research. It comprises eminent physicians, researchers, biostatisticians and officials that will scrutinize data for compliance with given patient safety standards as per Good Clinical Practices (GCP). It’s also vested with authority to decide on halting a study or an intervention arm until a subsequent review is conducted. It is supervised by the Chairman and each of the meeting is recorded by the designated Secretary.

In line with well-established editorial standards, the interim analyses will be done independently of the authors of the paper and investigators. To achieve that, NDSMB could establish an interim analysis sub-committee of independent individuals who will be named in acknowledgment section of the paper. The study statistician will be part of this committee and will give a written undertaking to keep that interim finding blind from the clinical investigators. The NDSMB meeting agenda could have an open and a closed part, the latter part will only by attended by interim analysis sub-committee. All decisions including termination of the study and related observations will be communicated to the investigators in writing.

### Patient Safety:

Diagnostics such as Electrocardiogram, Hepatotoxicity, Nephrotoxicity, Myelotoxicity is indicated once ADR is suspected and explained on Page 8 (Methods). Any additional incident or finding defined as adverse event (AE) during recruitment period will be reported to IT module. An adverse event (AE) is “any untoward medical occurrence in a patient or clinical investigation subject administered a pharmaceutical product that does not necessarily have a causal relationship with this treatment. For example, a physical injury resulting from fall or slip, a natural calamity during in-patient stay etc.” Investigators are also trained to ensure safe and secure environment for participants to provide necessary psychosocial support, when indicated.

### Audits:

The NDSMB can convene under its chairman at any time to independently review and audit findings and provide subsequent guidance on remaining conduct of the study. Its composition ensures that any conflicts of interests are avoided and patient safety and protection are prioritized.

### Patient and public involvement:

Because of the emergency nature, there was no opportunity to engage patients or their representative.

### Ethics Approval:

The proposed study is approved by National Bioethics Committee of Pakistan (attached separately as additional file**)** and IRBs at majority of participating sites including the lead institution, University of Health Sciences Lahore (attached separately as additional file**)**. Remaining sites have endorsed the ethics approval of both University of Health Sciences Lahore and National Bioethics Committee (NBC).

The approval from NBC is binding on primary investigators to keep it informed of any modifications in protocol should they arise in view of any of the factors including but not limited to emerging science or knowledge of disease that was previously unknown, clinical feedback or from participating patient(s), or subsequent to NDSMB review. Subsequent to any approval of protocol modifications, corresponding study investigators will be responsible for re-training or other changes to be communicated to each of the site.

No participant could be randomized without obtaining informed consent. It is the responsibility of the investigator to clearly communicate to potential participants the study objectives and lack of any definitive treatment of SARS-Cov-2. All investigators are trained GCP guidelines have been incorporated in the training module on informed consent so that investigators encourage potential participants to seek any clarification regarding study drugs, expected benefits and harms. In case written informed consent was not obtained due to clinical practices that bar use of paper in isolation area or obtained via phone, the circumstances and justification has to be mentioned in case reports Contact details of all patients must be maintained as a separate dataset for auditors to verify the compliance with protocol. A printed version of informed consent form developed in local language for this study has been delivered to each site in enough numbers (attached separately in email). For isolation areas allowing the use of printed copies, maintaining a separate folder for informed consent of all enrollees has been advised.

The protocol has been designed as an emergency measure to inform clinical effectiveness of off-label drugs in a resource-deficient settings. No biological specimen will be stored for any ancillary studies.

### Data Privacy:

All printed data (informed consent, CRF, diagnostic reports, death certificates) will be maintained in individual case files. All printed data will be secured in a separate storage area under strict custody of the site lead researcher. The electronic record of enrollment will identify enrollees as unique identification numbers generated at the time of randomization. All participants will be informed that the study data will be maintained for the period of one year and will be destroyed afterwards in all forms - electronic and paper.

### Data maintenance and dissemination:

NDSMB will be the custodian of the final trial data and investigators will give an undertaking for not using it in part of whole for any purpose without prior written authorization from the NDSMB.

The study is being conducted in view of lack of treatment options for mild to moderately symptomatic SARS-Cov-2 with a commitment to no-harm practices and a robust adverse drug reaction monitoring framework. Therefore, no liability exists with investigators.

Subsequent to NDSMB review and approval, findings of interim analysis and relevant results are to be announced publicly and submitted for publication in a scientific journal for dissemination to the scientific community and public at large as represented by the study participants.^23^ Any part or whole of the protocol, site data, or the entire dataset could be made available for academic use only.

### Authorship:

The final manuscript would be published as group authorship with investigators’ names mentioned.

### Declarations:

***Ethics Approval:*** The proposed study is approved by the National Bioethics Committee of Pakistan on April 22, 2020 (reference No.4-87/NBC-471-COVID-19-05/20/) and IRBs at most of the participating sites. Remaining sites have endorsed the ethics approval of both University of Health Sciences Lahore and National Bioethics Committee (NBC). Participants’ informed consent is intended to be sought on printed proforma designed in local language and no individual under the age of 18 years is to be enrolled. The approval letter is attached as supplemental material (NBC.pdf).

Certified that this trial has received ethical approval from the appropriate ethical committee as described above

### Strengths and Limitations of this study:

• By providing effectiveness data on three drugs used on patients with mild COVID-19, this multicenter randomized controlled will inform the treatment protocol so that hospital resources can be better utilized for patients with more severe symptoms. • It was commenced to immediately respond to the pandemic at a time when very little was known about the disease. There was no definitive treatment or effective vaccine available either. • Vaccine is not the substitute of COVID treatment since vaccines do not provide protection among all those inoculated. Therefore, studies on pharmaceutical management of mild COVID are needed.

## ATTACHMENTS (Supplementary files with 1^st^ submission in October 2021):

1. Case report form 2. Flow Diagram 3. Approval of NBC 4. Approval of UHS ERC. ERC, available from corresponding author on request.
